# Supplementary material for: SUFI: an automated approach to spectral unmixing of fluorescent multiplex images captured in mouse and post-mortem human brain tissues
Source: BMC Neurosci. 2023 Jan 25;24:6. doi: 10.1186/s12868-022-00765-1 (PMC9878864; doi:10.1186/s12868-022-00765-1)
Supplement: Supplementary file 1 — Additional file 1: Figure S1. Extracted spectral signatures (fingerprints) from single positive vs. multiplex lambda stacks. Comparison of spectral signatures extracted from single positives vs. a multiplex lambda stack using the Vector Component Analysis (VCA). In each subplot, normalized pixel intensity is plotted against wavelength. (i) solid lines represent the fingerprints extracted using single positives. (ii) dotted lines represent fingerprints extracted from a multiplex lambda stack. The color corresponds to peak wavelength for DAPI and Opal dyes. Lipofuscin is pseudo-colored to black. Root mean squared error (RMSE) between the two lines is calculated for each set of fingerprints. Figure S2. Extracted spectral signatures (fingerprints) of immunofluorescence data from post-mortem human brain tissue sections derived from a donor with Alzheimer's disease. Spectral signatures are extracted from single positive lambda stacks using the Vector Component Analysis (VCA). In each subplot, normalized pixel intensity is plotted against wavelength. The color corresponds to peak wavelength for DAPI and Opal dyes. Lipofuscin is pseudo-colored to black. [file 12868_2022_765_MOESM1_ESM.docx]

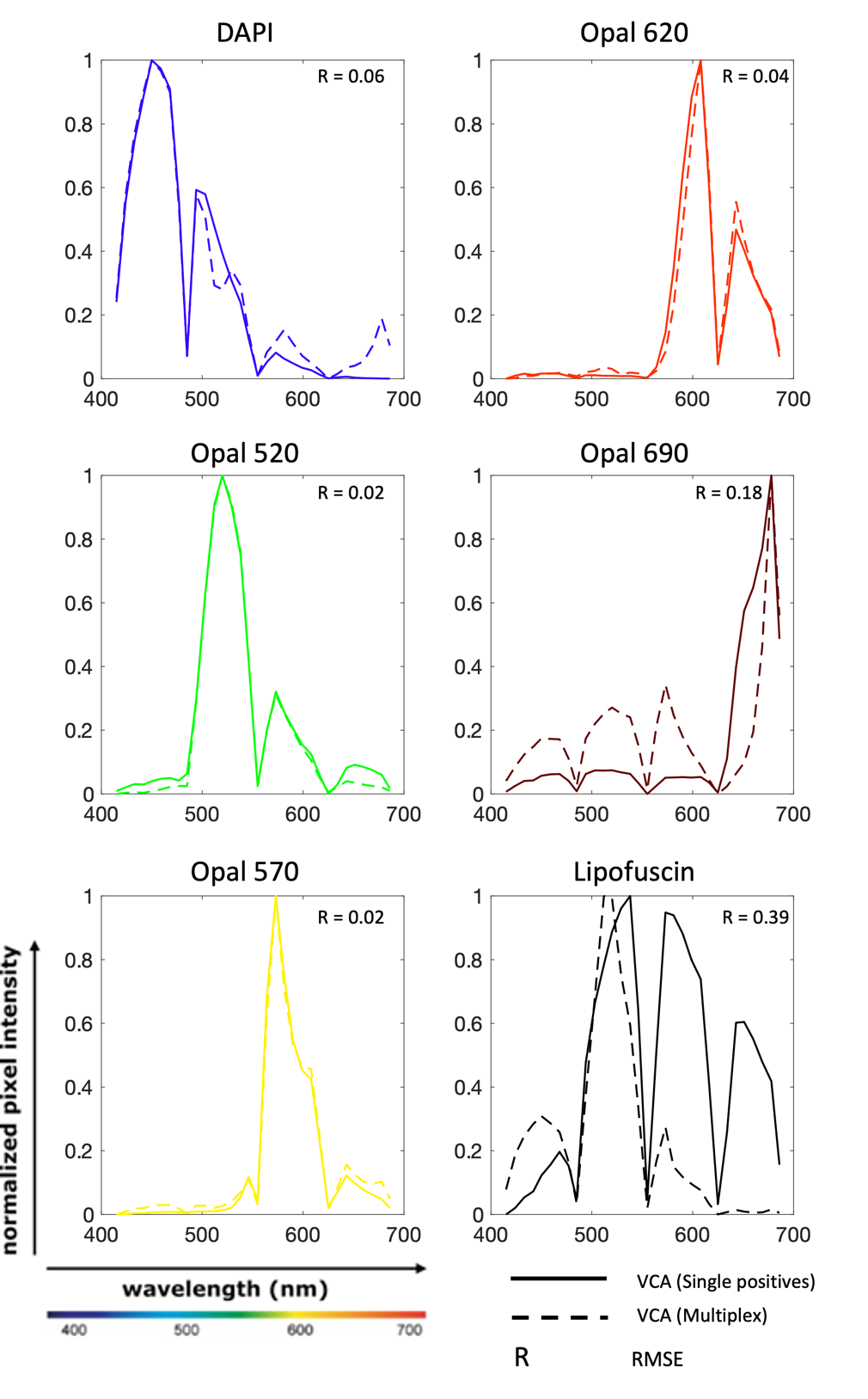


**Figure S1: Extracted spectral signatures (fingerprints) from single positive vs. multiplex lambda stacks.** Comparison of spectral signatures extracted from single positives vs. a multiplex lambda stack using the Vector Component Analysis (VCA). In each subplot, normalized pixel intensity is plotted against wavelength. (i) solid lines represent the fingerprints extracted using single positives. (ii) dotted lines represent fingerprints extracted from a multiplex lambda stack. The color corresponds to peak wavelength for DAPI and Opal dyes. Lipofuscin is pseudo-colored to black. Root mean squared error (RMSE) between the two lines is calculated for each set of fingerprints.


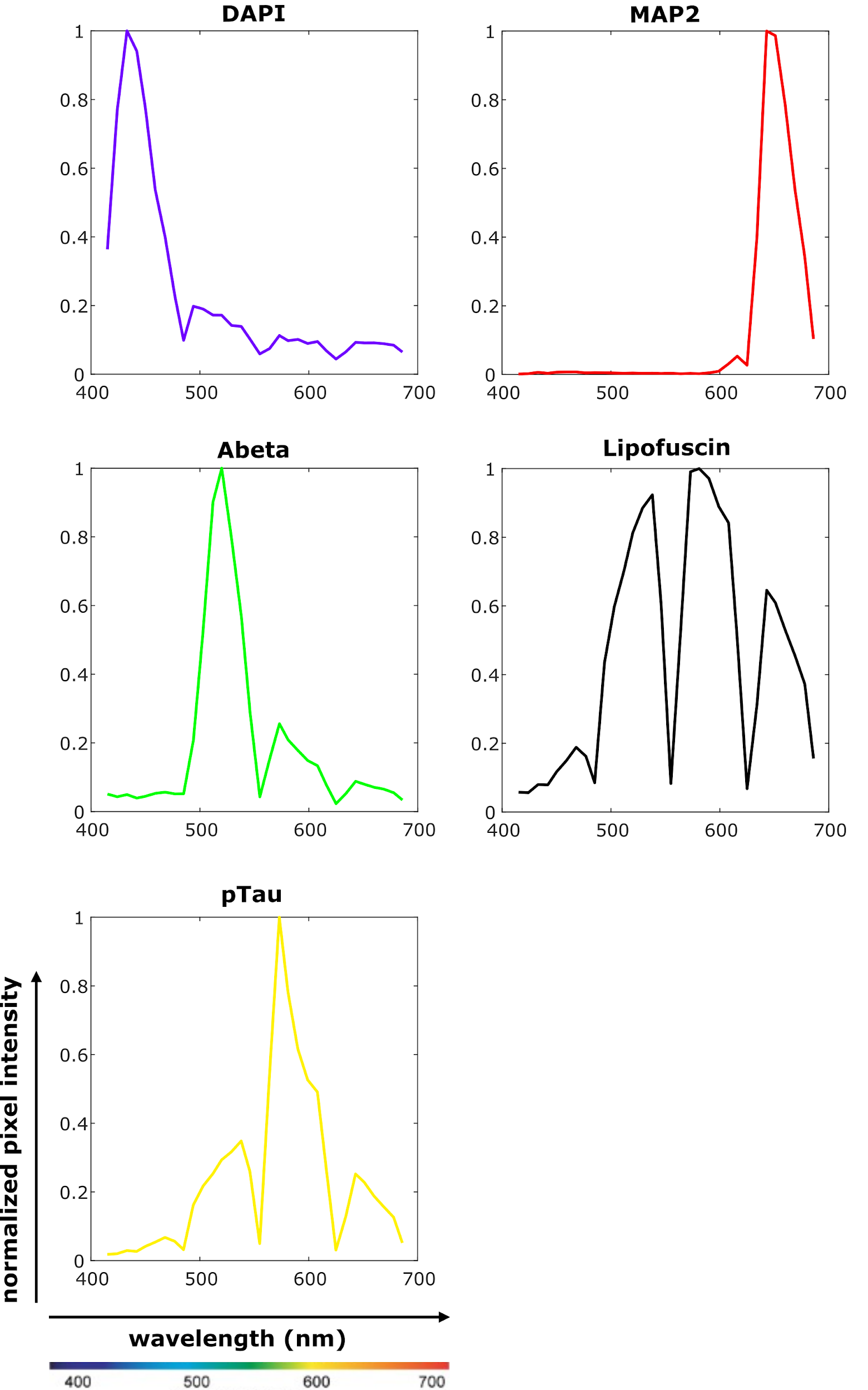


**Figure S2: Extracted spectral signatures (fingerprints) of immunofluorescence data from post-mortem human brain tissue sections derived from a donor with Alzheimer's disease.** Spectral signatures are extracted from single positive lambda stacks using the Vector Component Analysis (VCA). In each subplot, normalized pixel intensity is plotted against wavelength. The color corresponds to peak wavelength for DAPI and Opal dyes. Lipofuscin is pseudo-colored to black.
